# Differential associations between childhood maltreatment types and borderline personality disorder from the perspective of emotion dysregulation

**DOI:** 10.1186/s40479-023-00210-7

**Published:** 2023-02-06

**Authors:** Yan Yuan, Hyunji Lee, Christina E. Newhill, Shaun M. Eack, Rachel Fusco, Lori N. Scott

**Affiliations:** 1 School of Social Work, University of Pittsburgh, 2203 Cathedral of Learning, 4200 Fifth Ave, Pittsburgh, PA 15260 USA; 2 College of Social Work, Florida State University, Tallahassee, USA; 3 School of Social Work, University of Georgia, Athens, USA; 4 Department of Psychiatry, University of Pittsburgh, Pittsburgh, USA

**Keywords:** Childhood maltreatment, Abuse, Childhood trauma, Emotion dysregulation, Borderline personality disorder

## Abstract

**Background:**

Borderline Personality Disorder (BPD) is characterized by pervasive instability in a range of areas including interpersonal relationships, self-image, and affect. Extant studies have consistently identified significant correlations between childhood maltreatment (CM) and BPD. While exploring this CM-BPD link, a number of cross-sectional studies commonly emphasize the role of emotion dysregulation (ED). A better understanding of the associations between BPD and (1) CM and (2) ED are essential in formulating early, effective intervention approaches, and in addressing varied adverse impacts.

**Methods:**

This cross-sectional study analyzed a subset of baseline data collected for a larger community-based longitudinal study. Given that our current focus on CM and ED, only those participants who completed the baseline CM assessment and ED measure (*N* = 144) were included for the primary analyses. We conducted stepwise multivariate linear models to examine the differential relationships between BPD features, ED, and multiple CM types. A path analysis with latent factors using the structural equation modeling (SEM) method was performed to test the indirect effect from CM to BPD features via ED.

**Results:**

Linear regression models revealed that only emotional abuse (relative to other trauma types) was significantly associated with high BPD features. The SEM, by constructing direct and indirect effects simultaneously, showed that (1) ED partially mediated the path from CM to BPD features; and (2) CM played an important role in which the direct effect remained significant even after accounting for the indirect effect through ED.

**Conclusions:**

Our results highlight a most consistent association between emotional abuse and BPD, indicating its unique role in understanding BPD features in the context of CM. Further, shame-related negative appraisal and ED were found critical when examining the association between CM and BPD, possibly providing promising treatment targets for future practices.

**Supplementary Information:**

The online version contains supplementary material available at 10.1186/s40479-023-00210-7.

## Background

Borderline Personality Disorder (BPD) is characterized by pervasive instability in a range of areas including interpersonal relationships, self-image, and affect. People with BPD often evidence marked impulsivity manifested in various contexts such as overspending, risky sex, substance use, and/or binge eating [1]. The median population prevalence of BPD ranges from 1.6% to as high as 5.9% [1]; nevertheless, Lenzenweger [2] concluded the general population prevalence to be approximately 1% based on an overview of international and national studies [3–5]. BPD is associated with many adverse psychosocial impacts, including impairment in interpersonal relationships and employment, excessive utilization of medical services, and marital distress and violence [6–9].

## Literature review

Extant studies have consistently identified significant correlations between CM and BPD [10–16]. Commonly reported CMs by adults with BPD in previous studies include emotional abuse, verbal abuse, physical abuse, sexual abuse, and neglect, with sexual abuse being most frequently associated with a diagnosis of BPD among adults [17]. Additional CM-related risk factors for BPD include caregivers’ failure to protect, denial of feelings, emotional withdrawal, and non-interpersonal CM.

While exploring this CM-BPD link, a number of cross-sectional studies focusing on adult populations commonly emphasize the role of emotion dysregulation (ED), specifically a high sensitivity to negative emotional stimuli [18–20]. ED usually concerns failure in targeting one or several of these cognitive/behavioral areas. For instance, an individual might lack awareness of their own emotions, be unable to activate or achieve a goal, and/or lack adaptive strategies to alter emotional processes [21]. In line with Linehan’s biosocial theoretical perspective of BPD, ED can manifest as (1) excessive sensitivity to negative emotional stimuli, (2) a high amplitude of emotional response, (3) and/or a slow return to baseline [18]. For example, Tyrka et al. (2009) suggested that a sensitivity to negative emotional stimuli is a central aspect of BPD symptomatology, and they found in a community sample of adults with childhood experiences of abuse and neglect were more likely to report symptoms of BPD than those without such childhood adversities [16]. Gratz et al. (2008), employing a sample of inner-city substance users, found a partial indirect effect via emotion dysregulation between CM and BPD status. Their work further identified emotional abuse as the only factor significantly associated with BPD status after controlling for other forms of abuse and negative affect [15].

In addition to an overall ED deficit, specific ED subconstructs (such as high sensitivity, intense emotions and slow return) were further explored by several studies, among which an elevated sensitivity to negative emotions has been consistently identified among BPD individuals who experienced CM. And shame, guilt and anger were most frequently reported negative emotions [15, 20, 22–27]. For instance, shame is consistently associated with an early experience of sexual abuse and results in a wide array of negative outcomes relevant for BPD symptomatology, including low self-esteem, negative self-appraisals, intolerance of disapproval and problematic interpersonal relationships [24, 25]. Likewise, persistent states of shame, guilt and anger were commonly reported among BPD individuals with CM experience [23, 24]. Further, anger has been noted among CM survivors, especially for those who later carry a diagnosis of PTSD. Finally, maladaptive regulation of those reported negative emotions was reported to be associated with several psychopathologies, including BPD [28].

## Aims and significance of the current study

Although past studies examined ED and CM concerns among BPD individuals, the potential differential associations between CM types and (a) ED, and CM and (b) BPD remain unclear. While ED is a core feature of BPD, it is reasonable to propose that ED problems and CM are distinct concepts which worth further examining. More meaningfully, a better understanding of the associations is essential in formulating early, effective intervention approaches, and in addressing varied adverse impacts on interpersonal relationships and employment, excessive utilization of medical services, and marital distress and violence. Knowledge of key factors such as ED and CM will potentially contribute to early identification of BPD traits. Improved knowledge in this aspect will in particular facilitate effective prevention and inform future practice.

In light of this, the aims of the current study are: (1) to examine the differential association of CM types (specifically, physical, sexual, and emotional abuse, and physical and emotional neglect) with BPD features, and ED constructs with BPD, and (2) to examine the direct and indirect relationships between CM and BPD, potentially through the third channel of ED. In addition to a general relationship between CM and BPD as suggested by previous literature, we hypothesize that (1) differential associations exist between (1) CM and BPD features: Specifically, in line with a large number of cross-sectional studies, sexual abuse may have a stronger association with BPD relative to other CM types, and (2) further there will be a significant indirect effect of CM through the channel of ED examined by structural equation modeling.

## Methods

### Participants and procedures

This cross-sectional study is a secondary analysis of a subset of baseline data collected for a larger community-based longitudinal study (Pittsburgh Girls Study [PGS]). The PGS involves 2450 girls (now women) who were initially recruited in 1999 and 2000 when they were ages 5 to 8 years old (see Keenan et al., 2010 for further details on PGS recruitment and study design). Participants for the sub-study, which focused on aggressive and self-harming behavior in young women, were identified from the larger PGS based on self-reports of recent aggressive behavior, suicidality, or self-injury (see [29] for additional details). A total of 166 young women were recruited and consented to participate in the sub-study. During initial assessments (baseline) of the sub-study, participants completed a battery of clinical interviews and self-report measures (see Measures section for details). Follow-up assessments (data were not presented here) occurred at 6- and 12-months, respectively, after the initial assessments (Tables 1 and 2).

**Table 1:** Demographic Characteristics of Study Participants (*N* = 144)

Variables	*n* (%)
Age: Mean at Wave 1 (Range)	21.51 (18.83-24.91)
Race/Ethnicity	
African American	101 (70.1)
White	40 (27.8)
Multiracial	3 (2.1)
Sexual orientation	
Heterosexual orientation	107 (74.3)
Bisexual orientation	23 (16)
Gay/lesbian/homosexual orientation	12 (8.3)
Not sure	2 (1.4)
Marital status	
Never married	134 (93.1)
Married/living with someone	10 (6.9)
Education level	
Grade 7 to 12 did not graduate high school	14 (9.7)
High school/HS equivalent	60 (41.7)
College (graduated 2-year or 4-year college/part college)	67 (46.5)
Graduate/professional school (completed/part graduate or professional school)	3 (2.1)
Employment status	
Homemaker	2 (1.4)
Did not work due to disability	2 (1.4)
Did not work	49 (34)
Worked full time	36 (25)
Worked part time	55 (38.2)

**Table 2:** Measures

	Item #	Cronbach’s 𝛼	*M*	*SD*	Skewness	Kurtosis	Range
CTQ	Total	𝛼=.92 (6 subscales with MN)	54.2	14.5	1.55	6.13	34-117
	Total	𝛼=.91 (5 subscales without <M)	46.3	16.2	1.27	5.09	25-111
PN	1	𝛼=.71					
	2						
	4						
	6						
	26						
EA	3	𝛼=.82					
	8						
	14						
	18						
	25						
EN	5	𝛼=.84					
	7						
	13						
	19						
	28						
PA	9	𝛼=.76					
	11						
	12						
	15						
	17						
MN	10	𝛼=.79					
	16						
	22						
SA	20	𝛼=.93					
	21						
	23						
	24						
	27						
Anger (STAXI)	Total	𝛼=.91 (30 items)	69.8	13.3	0.48	2.62	43-109
T-Anger	STAXI12	𝛼=.88					
	STAXI13						
	STAXI14						
	STAXI15						
	STAXI16						
	STAXI17						
	STAXI18						
	STAXI19						
	STAXI20						
Ang-Con	STAXI24	𝛼=.81					
	STAXI28						
	STAXI31						
	STAXI35						
	STAXI38						
	STAXI40						
Ang-Out	STAXI27	𝛼=.83					
	STAXI29						
	STAXI32						
	STAXI34						
	STAXI39						
	STAXI42						
	STAXI43						
Ang-In	STAXI25	𝛼=.71					
	STAXI26						
	STAXI30						
	STAXI33						
	STAXI36						
	STAXI37						
	STAXI41						
PAIBOR	Total	𝛼=.87	59.5	11.4	−0.07	2.49	32-85
Affect Instability	paibor1	𝛼=.72					
	paibor4						
	paibor7r						
	paibor10						
	paibor14r						
	paibor18						
Identity Problems	paibor2	𝛼=.68					
	paibor5						
	paibor8						
	paibor11						
	paibor15						
	paibor19r						
Negative Relationships	paibor3	𝛼=.64					
	paibor6						
	paibor9						
	paibor12r						
	paibor16						
	paibor20r						
Self-harm	paibor13	𝛼=.73					
	paibor17						
	paibor21						
	paibor22						
	paibor23						
	paibor24r						
GASP	Total	𝛼=.80	71.1	14.9	−0.48	2.76	27-104
Guilt-Negative-Behavior-Evaluation	1	𝛼=.69					
	9						
	14						
	16						
Guilt-Repair	2	𝛼=.54					
	5						
	11						
	15						
Shame-Negative-Self-Evaluation	3	𝛼=.72					
	6						
	10						
	13						
Shame-Withdraw	4	𝛼=.55					
	7						
	8						
	12						
DERS	Total	𝛼=.91	91.3	20.7	0.34	2.8	50-149
Nonacceptance of emotional responses	11	𝛼=.85					
	12						
	21						
	23						
	25						
	29						
Difficulty engaging in goal-directed behavior	13	𝛼=.72					
	18						
	20R						
	26						
	33						
Impulse control difficulties	3	𝛼=.81					
	14						
	19						
	24r						
	27						
	32						
Lack of emotional awareness	2r	𝛼=.85					
	6r						
	8r						
	10r						
	17r						
	34r						
Limited access to emotion regulation strategies	15	𝛼=85					
	16						
	22r						
	28						
	30						
	31						
	35						
	36						
Lack of emotional clarity	1r	𝛼=.76					
	4						
	5						
	7r						
	9						

Given that our current focus is on CM and ED, only those participants who completed the baseline assessment measures of CM and ED (*N* = 144) were included for the primary analyses and the results presented here. These participants were between the ages of 18 and 24 (*M* = 21.51, *SD* = 1.57), and were primarily African American or non-Hispanic White. The demographics of this sub-study sample were similar to those of the larger longitudinal study (redacted citation) from which participants were selected (see Table 1 for additional information).

### Measures

#### BPD in the linear regression models

The Structured Interview for DSM-IV-TR Personality (SIDP-IV [30];) was used to generate dimensional BPD scores for our linear models. The SIDP-IV is a semi-structured diagnostic interview for DSM-IV-TR personality disorders. Interviews were administered by research staff with a bachelor’s degree or higher who were trained to reliability by a doctoral-level clinical psychologist. SIDP-IV items are rated on a 0 to 3 scale (0 = *not present*, 1 = *subthreshold*, 2 = *present*, 3 = *strongly present*). Dimensional scores (a sum of all BPD item scores) were used as an index of BPD symptomatology severity. The BPD items demonstrated adequate internal consistency for dimensional BPD scores in this subsample (Cronbach’s *α* = .87).

#### BPD in the SEM

In addition to SIDP-IV, we used the Personality Assessment Inventory-Borderline Features Scale (PAI-BOR, [31]) in our structure equation model (SEM). PAI-BOR is a 24-item self-report measure that assesses four dimensions underlying BPD: affective instability, identity problems, negative emotions, and self-harm. The rational to use two BPD measures is that SIDP-IV is clinically-administered and can generate less biased dimensional diagnostic scores; whereas PAI-BOR contains four BPD domains which have been statistically validated and therefore can be conveniently used to specify the measurement model in SEM in addition to assess the relationships among three latent factors (ED, CM and BPD). Intraclass coefficients among this sample for subscales are as follows: Affect instability (*α* = .72), identity problems (*α = .*68), self-harm (*α = .*73), and negative relationships (*α =* .64).

#### CM

The Childhood Trauma Questionnaire Short Version (CTQ-SF [32];) items ask about experiences from early childhood to adolescence, which are rated on a 5-point scale with response options ranging from Never True to Very Often True. The CTQ-SF produces a total score and five CM-related subconstructs—physical, sexual, and emotional abuse, and physical and emotional neglect. The CTQ-SF showed good reliability among this sample. Intraclass correlation coefficients for subscales are: Physical neglect (*α =* .71), emotional abuse (*α =* .82), emotional neglect (*α =* .84), physical abuse (*α =* .76), and sexual abuse (*α =* .93).

#### Emotion dysregulation

The Difficulties in Emotion Regulation Scale (DERS [33];) is a 36-item self-report measure that was developed to assess emotion dysregulation comprehensively, including items that reflect difficulties in six emotional dimensions: Non-acceptance, Goals, Impulse, Strategies and Clarity [33]. More specifically, Non-acceptance means non-accepting reactions to negative emotions or stress; the Goals dimension contains items reflecting difficulties in engaging in goal-directed behaviors (such as concentrating or accomplishing tasks); the Impulse dimension consists of items that describe difficulties with controlling behaviors under negative emotions; the Awareness (reverse-coded) scale assesses the ability to attend to and recognize emotions; the Strategies dimension includes items that evaluate limited access to regulation strategies; and Clarity measures lack of clarity about one’s own emotions(e.g. unable to identify one’s emotions). Each item of the DERS is rated on a 5-point scale ranging from 1 “almost never” to 5 “almost always”. DERS demonstrated good internal consistency among our sample as indicated by intraclass correlation coefficients for subscales of non-acceptance (*α =* .85), goals (*α =* .72), impulse (*α =* .81), strategies (*α =* .85) and clarity (*α =* .85).

#### Shame/guilt

The Guilt and Shame Proneness scale (GASP) is a 16-item self-report scale that assesses individuals’ tendencies to experience shame and guilt following embarrassing or offensive events across different settings [34]. The GASP consists of two shame subscales (negative behavior-evaluations and repair action tendencies) and two guilt subscales (negative self-evaluations and withdrawal action tendencies). For the two guilt subscales, negative behavior-evaluations items address bad feelings about one’s actions, whereas repair items describe behavioral intentions such as correcting one’s mistakes (e.g., “you would try to act more considerately toward your friends”). As far as the shame subscales, negative self-evaluations consist of items about feeling bad about oneself, whereas withdrawal items address tendencies to hide from the public (e.g., “you would avoid the guests until they leave”). Each item of the GASP is rated on a 7-point scale, with “1″ indicating “very unlikely” and “7″ indicating “very likely”. Finally, internal consistency for GASP was unsatisfactory among our sample. The intraclass correlation coefficients are: Negative behavior-evaluations (*α* = .69), repair action tendencies (*α* = .54), negative self-evaluations (*α* = .72) and withdrawal action tendencies (*α* = .55).

#### Anger

The original State-Trait Anger Expression Inventory-2 (STAXI-2) is a 57-item self-report measure comprised of six subscales: State Anger, Trait Anger, Anger Expression-In, Anger Expression-Out, Anger Control-In, and Anger Control-Out [35]. We utilized an abbreviated anger scale that included only Trait Anger, Anger Expression-In, Anger Expression-Out, and Anger Control (we used mean scores of both Control-in and out scores, which were also reverse coded). In terms of each subscale, Trait Anger measures the disposition to experience anger with or without provocation; Anger Expression-In assesses the frequency of controlling one’s angry feelings; Anger Expression-Out measures how often one takes actions upon his/her anger; and Anger Control measures one’s ability to control one’s anger by utilizing positive outlets (Control-out) or calming oneself down (Control-in). The internal consistency of each subscale in this sample was adequate. The intraclass correlation coefficients are: Trait Anger (*α* = .88), Anger Expression-In (*α* = .71), Anger Expression-Out (*α* = .83), and Anger Control (*α* = .81).

### Analyses

#### Research question 1: linear regression models

To examine the differential relationships between BPD features, ED, and specific CMs, we conducted stepwise multivariate linear models. The initial model was comprised of five CM types as main predictors. Step two included DERS constructs as additional independent variables. Step three added four anger variables: Trait Anger, Anger Expression-out, Anger Expression-in and Anger Control (this variable was reversed coded). The final step further included four subconstructs of shame/guilt. Finally, performance of different models (e.g. model *R*^2^) were evaluated and compared.

#### Research requestion 2: SEM

**Fig. 1:**
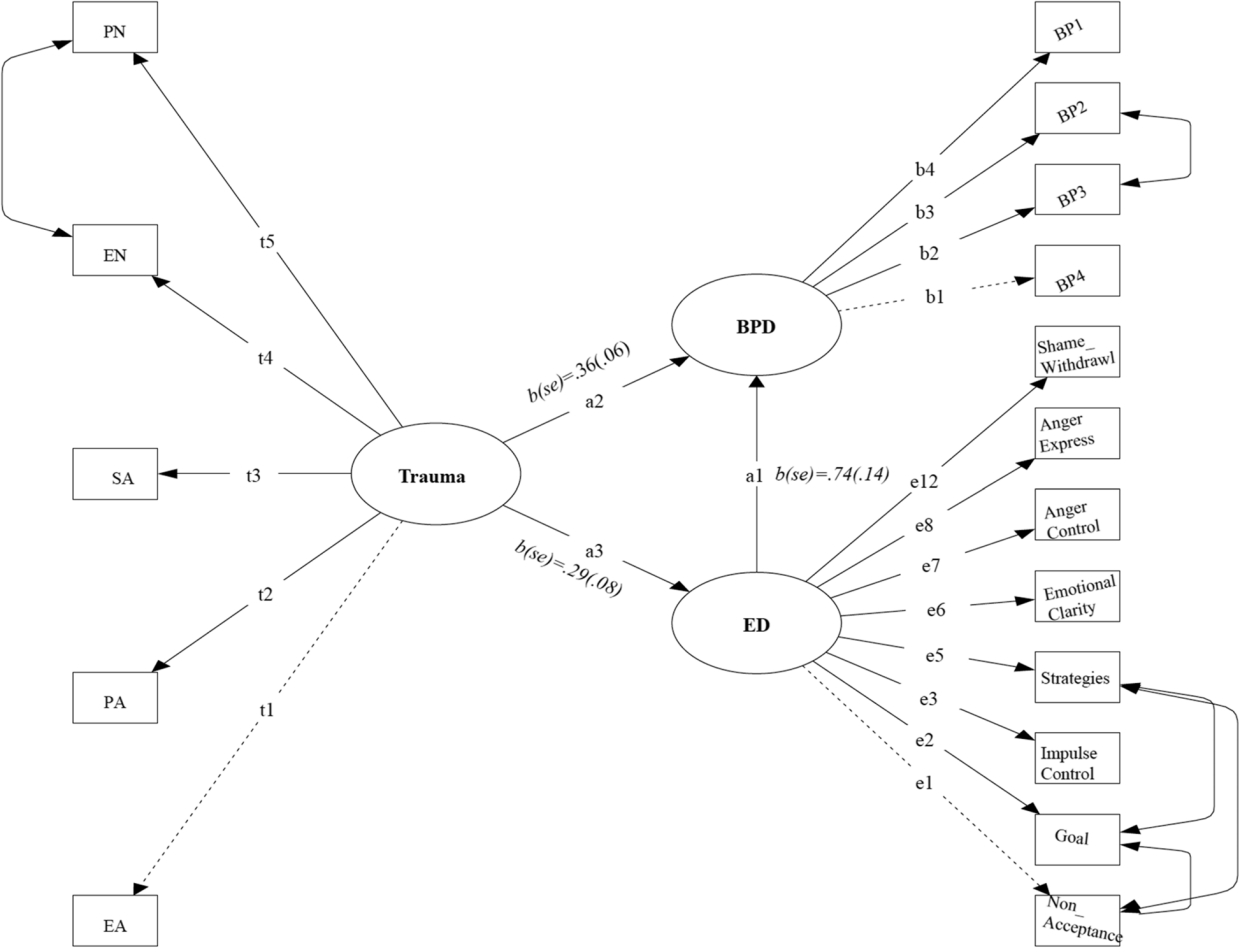
A Path Diagram of Trauma Predicting BPD Partially Mediated via Emotion Dysregulation Dimensions (PN=Physical Neglect, EN = Emotional Neglect, SA = Sexual Abuse, PA = Physical Abuse, EA = Emotional Abuse, BP 1 = Affective instability, BP 2 = Identity problems, BP 3 = Negative Relations, BP 4 = Self-harm. Measurement model parameters which were omitted here for a more clear and concise display. All parameters were significant except for three ED subconstructs. Parameters of the paths displayed via dotted lines were fixed. Double arrow lines stand for the covariances among subconstructs)

In order to test the indirect effect from CM to higher BPD features through ED, we conducted path analysis with latent factors using the structural equation modeling (SEM) method in R. The structural model was comprised of the latent predictor CM, the latent outcome variable BPD, and the mediator ED. The measurement model is specified as follows: CM is measured by five subtypes (physical abuse, sexual abuse, emotional abuse, physical neglect and emotional neglect), BPD by four symptomatic categories (affective instability, identity problems, negative emotions and self-harm), and ED by six emotional subconstructs (non-acceptance, goals, impulse, awareness, strategies, and clarity) (Fig. 1). Were model modifications needed, two methods will be used using packages of “aod” and “Rsolnp” in R [36, 37], specifically, (1) Wald statistics (estimated increase in X^2^ given a prior estimated path parameter fixed to a known value) and (2) LaGrange Multiplier method (predicted decrease in X^2^ given a prior fixed path parameter were to be estimated) [38]. Finally, a *p*-value equals or is less than .05 will be considered significant [39].

## Results

### Preliminary analyses

To select potential control variables, characteristic differences based on demographic factors (such as race, sexual orientation, marriage status, employment and education) in BPD dimensional scores were assessed using Multi-factor Analysis of Variance (ANOVA). Results from Multi-factor ANOVA evidenced no significant between-group differences in BPD scores.

### Regression models

Table 3 presents parameters and model fit indices of all our multiple regression models. Results from model 1 indicated that only emotional abuse (*b* = .19, *t* = 2.01, *p* = .05) was significantly associated with higher BPD features. The overall model *R*^2^ was significant, accounting for approximately 18% of the variance. Adding DERS subconstructs, model 2 showed that the effect of EA became marginally significant and impulsivity (*b* = .28, *t* = 3.60, *p* < .001) was significantly correlated with higher BPD scores. There is a significant increase in model *R,*^2^ indicating an improvement in model performance. In Model 3, we introduced four additional predictors: Trait Anger, Anger Expression-out, Anger Expression-in and Anger Control (this variable was reversed coded). There was no improvement in the model performance and impulsivity remained significant (*b* = .23, *t* = 2.38, *p* < .05) whereas other predictors were not. In the final model, four subconstructs of shame/guilt were added, and results demonstrated that EA (*b* = .17, *t* = 1.98, *p* < .001) and Shame (negative self-evaluation; *b* = − 0.79, *t* = − 2.49, *p* < .05) were significantly associated with BPD scores. The final model was significantly improved from model 3 and 4, accounting for about 41% of the variance. Finally, other types of CM, including physical and sexual abuse, and neglect were not significant across all our models.

**Table 3:** Regression Models Predicting BPD Features (*N* = 144)

Variables	Model 1				Model2				Model 3				Model 4			
	b	se	t	*p*	b	se	t	*p*	b	se	t	*p*	b	se	t	*p*
(Intercept)	0.96	1.11	0.86	0.39	−3.88	1.78	−2.18	0.03*	−4.46	2.84	−1.57	0.12	−4.30	3.49	−1.23	0.22
CTQ_EA	0.19	0.10	2.01	0.05*	0.15	0.09	1.76	0.08^a^	0.15	0.09	1.70	0.09^a^	0.17	0.09	1.98	0.05*
CTQ_PA	0.19	0.13	1.43	0.16	0.16	0.12	1.35	0.18	0.10	0.12	0.86	0.39	0.10	0.12	0.88	0.38
CTQ_SA	0.07	0.08	0.88	0.38	0.10	0.07	1.41	0.16	0.10	0.07	1.31	0.19	0.08	0.07	1.17	0.24
CTQ_EN	0.05	0.10	0.54	0.59	−0.02	0.09	−0.25	0.80	0.01	0.09	0.07	0.94	−0.04	0.09	−0.41	0.68
CTQ_PN	0.17	0.13	1.30	0.19	0.13	0.12	1.05	0.29	0.11	0.12	0.89	0.38	0.14	0.12	1.16	0.25
Non-acceptance					0.04	0.08	0.52	0.60	0.02	0.08	0.28	0.78	0.07	0.08	0.82	0.41
Goals					−0.14	0.10	−1.41	0.16	−0.15	0.10	− 1.52	0.13	− 0.11	0.10	− 1.10	0.28
Impulse					0.28	0.08	3.60	0.00***	0.24	0.10	2.38	0.02*	0.19	0.10	1.90	0.06^a^
Awareness					−0.03	0.08	−0.40	0.69	0.00	0.08	0.00	1.00	0.05	0.08	0.69	0.49
Strategies					0.07	0.08	0.92	0.36	0.06	0.08	0.67	0.50	0.10	0.08	1.20	0.23
Clarity					0.21	0.11	1.85	0.07^a^	0.20	0.12	1.68	0.10.	0.14	0.12	1.19	0.24
Trait Anger									0.27	0.81	0.34	0.74	0.49	0.81	0.60	0.55
Anger Control									−0.70	0.68	−1.04	0.30	−0.69	0.67	−1.02	0.31
Anger Expression Out									0.91	0.76	1.19	0.24	0.63	0.79	0.80	0.42
Anger Expression in									0.80	0.74	1.08	0.28	0.71	0.72	0.99	0.32
NBE													−0.08	0.34	−0.23	0.82
GR													0.64	0.41	1.57	0.12
NSE													−0.79	0.32	−2.49	0.01*
SW													−0.24	0.31	−0.79	0.43
*R2adjusted*	0.18				0.36				0.38				0.41			
*F*	7.47				8.45				6.88				6.22			
*df*	5138				11,132				15,128				19,124			
*p (∆R2)*					<.001***				0.1				0.05*			

***: *p*<.001; **: *p*<0.01; *p<.05

^a^is a marginal significance which is close to 0.05 but larger than 0.05

### SEM

The initial SEM model had unsatisfactory performance (*CFI* = .67, *SRMR* = .12, and *RMSEA* = .13 (90%*CI*: .11 ~ .14)). As post hoc procedures, subsequent modifications were performed using: (1) Wald statistics and (2) LaGrange Multiplier method [38]. The stepwise multivariate Wald test in Lavaan [40] indicated that four non-significant paths can be eliminated from the initial model (the predictions of ED by emotional awareness, both guilt subconstructs, and one shame subscale of negative self-evaluation). The LaGrange Multiplier method was subsequently applied for further diagnosis and modification. From the results, five covariances (See Fig. 1 and Table 4) were added iteratively to improve the model performance. In this procedure, only covariances underlying the same factor were selected iteratively (e.g., ED manifest variables were allowed to covary); whereas cross-loadings (variables measuring across factors: e.g., between ED subconstruct and CM subconstruct) were not allowed given that it will be theoretically misleading.

**Table 4:** Standardized parameter estimates for the SEM model

	***B***	***SE***	***z***	***p***	***ß***	***R***^***2***^
**Measurement Model**						
Trauma						
→ Childhood emotional abuse	1				.78	.61
→ Childhood physical abuse	.73	.09	8.13	.00	.77	.59
→ Childhood sexual abuse	.68	.12	5.66	.00	.52	.27
→ Childhood emotional neglect	.74	.11	6.64	.00	.61	.37
→ Childhood physical neglect	.46	.08	5.79	.00	.54	.29
ED						
→ Non-acceptance	1				.53	.28
→ Goal-directed behavior	.90	.15	6.13	.00	.59	.35
→ Impulse control	1.68	.26	6.36	.00	.84	.71
→ Emotional regulation strategies	1.68	.23	7.43	.00	.71	.50
→ Emotional clarity	.62	.14	4.35	.00	.45	.20
→ Anger control	−.10	.03	−3.99	.00	−.46	.21
→ Ange expression	.49	.08	6.19	.00	.79	.62
→ Shame-withdraw	.15	.04	3.65	.00	.36	.13
BPD						
→ Affective instability	1				.87	.75
→ Identity problems	.81	.09	8.55	.00	.69	.47
→ Negative emotions	.75	.09	8.15	.00	.65	.43
→ Self-harm	.72	.10	7.44	.00	.68	.47
**Structural Model**						
BPD						
→ Trauma (a2)	.29	.06	5.05	.00	.36	.82
→ ED (a1)	.83	.14	5.88	.00	.74	
ED						
→ Trauma (a3)	.21	.08	2.68	.01	.29	.08
Indirect Effect						
a1*a3	.17	.06	2.86	.00	.21	
Total Effect						
a2 + (a1*a3)	.46	.08	5.70	.00	.57	

The modified model was significantly improved from the initial model despite no significant difference from the observed model (*ΔX*^2^=367.57). However, the following indices showed an overall good fit of the final model [*CFI* = .93, *SRMR* = .067, and *RMSEA* = .06 (90%*CI*: .04 ~ .07)]. As can be seen in Table 4, the final model revealed that three factors were generally well identified with good construct validity. From information presented in Fig. 1 and Table 4, there were significant direct effects of CM on ED (*b* = .36, *z* = 5.05, *p* < .001) and ED on BPD (*b* = .74, *z* = 5.88, *p* < .001). After accounting for the indirect effect of CM on BPD via ED (*Δb* = .21, *z* = 2.86, *p* < .01), the total effect of CM on BPD remained significant (*b* = .57, *z* = 5.70, *p* < .001). In other words, higher CM significantly predicted heightened BPD symptomatology, partially mediated through ED. In addition, CM showed a significant and unique effect after controlling for the indirect effect via ED.

## Discussion

### Summary of key findings

By testing direct associations between BPD features, ED constructs and CM types, we have identified that only emotional abuse (relative to other CM types) was significantly associated with high BPD features; further, some ED constructs (such as impulsivity and shame-related negative appraisals) may bear special meanings to BPD features. Our SEM model, by constructing direct and indirect effects simultaneously, further revealed that (1) ED partially mediated the path from CM to BPD features; and (2 the direct effect of CM remained significant even after accounting for the indirect effect through ED.

### CM types and emotional abuse

Although multiple regression results evidenced a significant effect of emotional abuse (EA) on BPD symptomatology, other types of abuse were not significantly associated with BPD features. Emotional abuse significantly predicted BPD features, replicating previous findings on the relationship between childhood emotional abuse and BPD symptoms [41–46]. Commonly posited etiological explanations for this finding have included the presence of emotion dysregulation, attachment disturbance and a dynamic biosocial interaction [41, 42, 47, 48].

Indeed, this finding on emotional abuse could be interpreted to be consistent with Linehan’s biosocial theory, in which BPD etiology is conceptualized as a dynamic interplay between inherited emotion regulation vulnerabilities and invalidating environments [18]. Examples of emotional abuse items in the CTQ are: “People in my family called me things like stupid, lazy, or ugly” and “I thought my parents wished I had never been born” [32]. Those verbal assaults are typical of invalidating environments, where belittling of feelings, and suppression of negative emotions frequently happen. Therefore, emotional abuse may be a key feature of invalidating environments that elevates risk for BPD symptoms. Likewise, Rosenstein et al. (2018) posit that emotional abuse is typical of invalidating environment, which accounts for BPD symptomatology [49]. More recent empirical studies also lend support to this line of finding by showing evidence that emotional abuse independently predicts heightened BPD features when controlling for other types of trauma [15, 42, 49], which was specifically related to ED problems (a core BPD characteristic). For instance, from the perspective of ED, researchers suggested that BPD individuals might have an inherited tendency to over-regulate negative emotion to adapt to such invalidating environments.

Inconsistent with a large body of BPD literature, our findings did not support a significant effect of sexual abuse on BPD symptomatology [43, 50–52]. Notably, our participants endorsed the lowest mean score of sexual abuse (*Mean* = 7.56, *SD* = 5.14) relative to other CM types. The comparatively low mean score may be partially explained by the fact that emotional abuse and physical neglect are more prevalent than sexual abuse in the general population [50, 53, 54]. Hence, given the lower rates of sexual abuse in our sample than those in clinical samples, it might be possible that we may not have found a relationship due to limited range on this variable.

### Shame and emotion dysregulation

A subtype of shame was shown to have a significant association with BPD features. Specifically, this subtype is a negative self-evaluation which denotes an unfavorable appraisal of self as a result of feeling shame. This interesting finding is in line with attachment theoretical explanations. Griffin and Bartholomew [55] conceptualize adult attachment styles in terms of mental representations of self and others as follows: positive self and other representations (secure pattern), positive self and negative other representations (dismissing pattern), negative self and positive other representations (preoccupied pattern), and negative self and other representations (fearful pattern). From this perspective, shame-negative-self is analogous to negative-self dimension. The function of this particular emotion might resemble that of a preoccupied anxious attachment, which has been consistently marked among people with CM exposure and those with BPD [47, 56, 57]. Further, shame is an example of social emotions, which primarily arise within interpersonal contexts [58]. Socially maladaptive regulation of shame can undermine one’s abilities to manage interpersonal relationships and vice versa. Such interrelatedness between interpersonal context and social emotions, hence, is highly compatible with BPD symptomatology.

Finally, our SEM results supported a partial indirect effect of CM on BPD features via elevated ED. This corroborates multiple lines of BPD literature [15, 59–61]. Further, there were studies that investigated how unique aspects of emotion dysregulation might be differentially associated with distinct CM types in accounting for higher BPD features. Researchers found that emotional neglect was related to less adaptive emotion regulation abilities (e.g., less frequent use of cognitive reappraisal), whereas emotional abuse was associated with higher dysfunctional or maladaptive emotion regulation strategies (more frequent use of expressive suppression). Although precise definitions of emotional neglect vary by state laws, emotional neglect is commonly defined as the failure of a parent or caretaker to provide affection or emotional support to the child [62]. Emotional neglect also includes any act that places the child at risk of being exposed to parental substance abuse or domestic violence [62]. These unique influences from particular aspects of ED did not emerge for other CM types [41, 63]. It is noteworthy that the effect of CM remained significant even after accounting for ED, indicating a unique role of CM in exacerbating BPD symptoms that is worthy of further investigation.

### Limitations, strengths and moving forward

One limitation of our study concerns the use of self-report measures (with the exception of our interview measure of BPD features), which can lead to recall biases. In terms of participants, our sample included only females (though the sample is diverse with regard to race and socioeconomic status); hence, generalizability to other genders is limited. Further, our age range is restricted to emerging adulthood, hence generalizability to other developmental stages can be limited. Finally, our study utilizes cross-sectional design, and thus our results only suggest correlations and statistical mediation.

More rigorous designs will be required to obtain more reliable knowledge. For example, future research should address comparing the differential effects between momentary emotional reactions and stable traits in exacerbating BPD symptoms after traumatic exposure in order to gain more knowledge about the specifics of ED. Moreover, different age groups can be recruited (such as adolescents and adults in the late twenties) and members of different racial/ethnic groups to further advance the current knowledge on different developmental ages and the role of culture. In addition, research studies can utilize repeated measures and causal inference techniques to improve the research design.

Despite the limitations, we comprehensively investigated ED, several distinct forms of NA and unique CM types in affecting BPD symptoms during emerging young adulthood. We revealed that emotional abuse in relative to other CM types can be specifically related to BPD features, and trainings on regulating CM-related social emotions, such as shame, can be a potential target for future practice.

## Conclusions

Our results highlight a most consistent association between emotional abuse and BPD, indicating its unique role in understanding BPD features in the context of childhood maltreatment. Further, shame-related negative appraisal and ED were found critical when examining the association between CM and BPD, possibly providing promising treatment targets for future practices.

First, early screening of CM-related symptoms and employment of trauma-informed care should be integrated into traditional BPD treatments and in settings where individuals with BPD who are in crisis may be seen, e.g. psychiatric emergency departments and outpatient/inpatient care units. Second, emotional regulation difficulties should be targeted when treating people with CM experiences. Third, it can be especially useful to address key CM-related negative emotions in treatment, such as shame and related maladaptive regulating strategies.

Regarding CM-related care relevant to BPD populations, as inspired by our study, a trauma-informed emotion regulation skills training can potentially include topics such as (1) mindfulness strategies for coping with CM-related emotions, (2) validation of negative emotions, and (3) learning reappraisal of negative experiences. Furthermore, for individuals with BPD without a diagnosis of CM or stress-related disorders, facilitating a supportive, genuine and empathic dialogue at minimum would promote early and accurate screening for CM symptomatology. As noted earlier, emotional abuse and emotion-related invalidation are highly prevalent among BPD populations (with or without a co-occurring trauma-related diagnosis); therefore, emotion regulation skills training targeting emotional invalidation can potentially lead to effective results.

For those with active co-occurring diagnoses, trauma informed treatment work which integrates traditional BPD psychotherapies with trauma-processing narratives, psychoeducation sessions, and exposure-based techniques can be helpful [64–68]. Last but not least, maintaining control of therapy-interfering or other high-risk behaviors, e.g. self-harm, can be critical before implementing any type of trauma care or related treatments. Crises such as high levels of life-threatening (e.g., suicide attempts) and/or therapy-interfering behaviors (e.g., dishonesty with therapist, frequent threatening to quit or non-completion of any homework assigned) before processing traumatic memories and emotions, given that the presence of aforementioned crises might prevent the individuals from effectively discussing and managing emotion about the CM, or they may not have the skills yet to regulate the emotions. To this aim, it will be necessary to conduct an early evaluation of the risks, establish a trusting therapeutic relationship as well as develop action plans to ensure safety [69].

## Supplementary Information

**Additional file 1: Table 1.** Demographic Differences in BPD Scores (BOR). **Table 2.** Demographic Differences in BPD Scores (PAIBOR).

## Data Availability

The data that support the findings of this study are available from Pittsburgh Girls’ Study, but restrictions apply to the availability of these data, which were used under license for the current study, and so are not publicly available. Data are however available from the authors upon reasonable request and with permission of PI of Pittsburgh Girls’ Study.

## Abbreviations

CM: Childhood Maltreatment
ED: Emotion dysregulation
EA: Emotional abuse
BPD: Borderline Personality Disorder
PGS: Pittsburgh Girls Study
NA: Negative affect
SIDP-IV: Structured Interview for DSM-IV-TR Personality
PAI-BOR: Personality Assessment Inventory-Borderline Features Scale
CTQ-SF: Childhood Trauma Questionnaire Short Version
DERS: Difficulties in Emotion Regulation Scale
GASP: The Guilt and Shame Proneness scale
STAXI-2: State-Trait Anger Expression Inventory-2
SEM: Structural equation modeling

## References

[1] Association AP. Diagnostic and statistical manual of mental disorders (DSM-5®). Washington, D.C: American Psychiatric Pub; 2013.

[2] Lenzenweger MF (2010). Current status of the scientific study of the personality disorders: an overview of epidemiological, longitudinal, experimental psychopathology, and neurobehavioral perspectives. J Am Psychoanal Assoc.

[3] Coid J, Yang M, Tyrer P, Roberts A, Ullrich S (2006). Prevalence and correlates of personality disorder in Great Britain. Br J Psychiatry.

[4] Lenzenweger MF, Lane MC, Loranger AW, Kessler RC (2007). DSM-IV personality disorders in the National Comorbidity Survey Replication. Biol Psychiatry.

[5] Torgersen S, Kringlen E, Cramer V (2001). The prevalence of personality disorders in a community sample. Arch Gen Psychiatry.

[6] Gunderson JG. Borderline personality disorder: ontogeny of a diagnosis. Am J Psychiatry. 2009;166(5):530–9. 10.1176/appi.ajp.2009.08121825 PMC3145201 19411380

[7] Juurlink TT, Vukadin M, Stringer B, Westerman MJ, Lamers F, Anema JR (2019). Barriers and facilitators to employment in borderline personality disorder: a qualitative study among patients, mental health practitioners and insurance physicians. PLoS One.

[8] Lieb K, Zanarini MC, Schmahl C, Linehan MM, Bohus M (2004). Borderline personality disorder. Lancet.

[9] Whisman MA, Schonbrun YC (2009). Social consequences of borderline personality disorder symptoms in a population-based survey: marital distress, marital violence, and marital disruption. J Disord.

[10] Herman JL, Perry JC, van der Kolk BA. Childhood trauma in borderline personality disorder. Am J Psychiatry. 1989;146(4):490–5. 10.1176/ajp.146.4.490. 10.1176/ajp.146.4.490 2929750

[11] Machizawa-Summers S (2007). Childhood trauma and parental bonding among Japanese female patients with borderline personality disorder. Int J Psychol.

[12] Venta A, Kenkel-Mikelonis R, Sharp C. A preliminary study of the relation between trauma symptoms and emerging BPD in adolescent inpatients. Bull Menn Clin. 2012;76(2):130–46. 10.1521/bumc.2012.76.2.130. 10.1521/bumc.2012.76.2.130 22686392

[13] Weaver TL, Clum GA (1993). Early family environments and traumatic experiences associated with borderline personality disorder. J Consult Clin Psychol.

[14] Westphal M, Olfson M, Bravova M, Gameroff MJ, Gross R, Wickramaratne P (2013). Borderline personality disorder, exposure to interpersonal trauma, and psychiatric comorbidity in urban primary care patients. Psychiatry.

[15] Gratz KL, Tull MT, Baruch DE, Bornovalova MA, Lejuez CW. Factors associated with co-occurring borderline personality disorder among inner-city substance users: the roles of childhood maltreatment, negative affect intensity/reactivity, and emotion dysregulation. Compr Psychiatry. 2008;49(6):603–15. 10.1016/j.comppsych.2008.04.005 18970909

[16] Tyrka AR, Wyche MC, Kelly MM, Price LH, Carpenter LL (2009). Childhood maltreatment and adult personality disorder symptoms: influence of maltreatment type. Psychiatry Res.

[17] Yuan Y, Lee H, Newhill C, Eack S. A systematic review of the association between early childhood trauma and borderline personality disorder. J Pers Disord. 2023;37(1). 10.1521/pedi.2023.37.1.16 36723424

[18] Linehan M. Cognitive-behavioral treatment of borderline personality disorder. New York: Guilford Press; 1993.

[19] Lynch TR, Chapman AL, Rosenthal MZ, Kuo JR, Linehan MM (2006). Mechanisms of change in dialectical behavior therapy: theoretical and empirical observations. J Clin Psychol.

[20] Wagner AW, Rizvi SL, Harned MS (2007). Applications of dialectical behavior therapy to the treatment of complex trauma-related problems: when one case formulation does not fit all. J Trauma Stress.

[21] Gross JJ (2013). Handbook of emotion regulation, second edition [internet].

[22] Carpenter RW, Trull TJ (2013). Components of emotion dysregulation in borderline personality disorder: a review. Curr Psychiatry Rep.

[23] Cheavens JS, Rosenthal MZ, Daughters SB, Nowak J, Kosson D, Lynch TR (2005). An analogue investigation of the relationships among perceived parental criticism, negative affect, and borderline personality disorder features: the role of thought suppression. Behav Res Ther.

[24] Badour CL, Resnick HS, Kilpatrick DG. Associations between specific negative emotions and DSM-5 PTSD among a national sample of interpersonal trauma survivors. J Interpers. 2017;76(4):1620–41 Violence. 10.1177/0886260515589930 PMC4769114 26088902

[25] Peters JR, Geiger PJ (2016). Borderline personality disorder and self-conscious affect: too much shame but not enough guilt?. Personal Disord.

[26] Rusch N, Schulz D, Valerius G, Steil R, Bohus M, Schmahl C (2011). Disgust and implicit self-concept in women with borderline personality disorder and posttraumatic stress disorder. Eur Arch Psychiatry Clin Neurosci.

[27] Amstadter AB, Vernon LL (2008). J Aggress Maltreatment Trauma.

[28] Glück T, Knefel M, Lueger-Schuster B (2017). A network analysis of anger, shame, proposed ICD-11 post-traumatic stress disorder, and different types of childhood trauma in foster care settings in a sample of adult survivors. Eur J Psychotraumatol.

[29] Scott LN, Wright AG, Beeney JE, Lazarus SA, Pilkonis PA, Stepp SD (2017). Borderline personality disorder symptoms and aggression: a within-person process model. J Abnorm Psychol.

[30] Pfohl B, Blum N, Zimmerman M (1997). Structured interview for DSM-IV personality: Sidp-IV.

[31] Morey LC. Personality assessment inventory. Odessa: Psychological Assessment Resources; 1991.

[32] Bernstein DP, Stein JA, Newcomb MD, Walker E, Pogge D, Ahluvalia T (2003). Development and validation of a brief screening version of the Childhood Trauma Questionnaire. Child Abuse Negl.

[33] Gratz KL, Roemer L (2004). Multidimensional assessment of emotion regulation and dysregulation: development, factor structure, and initial validation of the difficulties in emotion regulation scale. J Psychopathol Behav Assess.

[34] Cohen TR, Wolf ST, Panter AT, Insko CA (2011). Introducing the GASP scale: a new measure of guilt and shame proneness. J Pers Soc Psychol.

[35] Spielberger CD (1999). State-trait anger expression inventory-2.

[36] Ghalanos A, Theussl S. Rsolnp: general non-linear optimization using augmented lagrange multiplier method; 2015.

[37] Lesnoff M, Lancelot R (2012). aod: analysis of overdispersed data.

[38] Mueller RO, Hancock GR. Structural equation modeling. In Hancock GR, Stapleton LM, Mueller RO. (Eds.), The reviewer’s guide to quantitative methods in the social sciences. Routledge/Taylor & Francis Group. 2019. p. 445–56. 10.4324/9781315755649-33.

[39] Yates F, Mather K (1963). Ronald Aylmer Fisher, 1890-1962.

[40] Rosseel Y. lavaan: an R package for structural equation modeling. University of California at Los Angeles. J Stat Softw. 2012;48:1–36.

[41] Carvalho Fernando S, Beblo T, Schlosser N, Terfehr K, Otte C, Lowe B (2014). The impact of self-reported childhood trauma on emotion regulation in borderline personality disorder and major depression. J Trauma Dissociation.

[42] Kuo JR, Khoury JE, Metcalfe R, Fitzpatrick S, Goodwill A (2015). An examination of the relationship between childhood emotional abuse and borderline personality disorder features: the role of difficulties with emotion regulation. Child Abuse Negl.

[43] Laporte L, Paris J, Guttman H, Russell J (2011). Psychopathology, childhood trauma, and personality traits in patients with borderline personality disorder and their sisters. J Disord.

[44] Wota AP, Byrne C, Murray I, Ofuafor T, Nisar Z, Neuner F (2014). An examination of childhood trauma in individuals attending an adult mental health service. Ir J Psychol Med.

[45] Zhang TH, Chow A, Wang LL, Yu JH, Dai YF, Lu X (2013). Childhood maltreatment profile in a clinical population in China: a further analysis with existing data of an epidemiologic survey. Compr Psychiatry.

[46] Porter C, Palmier-Claus J, Branitsky A, Mansell W, Warwick H, Varese F (2020). Childhood adversity and borderline personality disorder: a meta-analysis. Acta Psychiatr Scand.

[47] Fossati A, Gratz KL, Somma A, Maffei C, Borroni S (2016). The mediating role of emotion dysregulation in the relations between childhood trauma history and adult attachment and borderline personality disorder features: a study of Italian nonclinical participants. J Disord.

[48] Laporte L, Paris J, Guttman H, Russell J, Correa JA (2012). Using a sibling design to compare childhood adversities in female patients with BPD and their sisters. Child Maltreat.

[49] Rosenstein LK, Ellison WD, Walsh E, Chelminski I, Dalrymple K, Zimmerman M (2018). The role of emotion regulation difficulties in the connection between childhood emotional abuse and borderline personality features. Personal Disord Theory Res Treat.

[50] Hengartner MP, Ajdacic-Gross V, Rodgers S, Müller M, Rössler W. Childhood adversity in association with personality disorder dimensions: new findings in an old debate. Eur Psychiatry. 2013;28(8):476–82. 10.1016/j.eurpsy.2013.04.004 23835016

[51] Hong PY, Lishner DA. General invalidation and trauma-specific invalidation as predictors of personality and subclinical psychopathology. Personal Individ Differ. 2016;89:211–6.

[52] Pagura J, Stein MB, Bolton JM, Cox BJ, Grant B, Sareen J. Comorbidity of borderline personality disorder and posttraumatic stress disorder in the U.S. population. J Psychiatr Res. 2010;44(16):1190–8. 10.1016/j.jpsychires.2010.04.016 PMC4209725 20537660

[53] Afifi TO, Mather A, Boman J, Fleisher W, Enns MW, Macmillan H, et al. Childhood adversity and personality disorders: results from a nationally representative population-based study. J Psychiatr Res. 2011;45(6):814–22. 10.1016/j.jpsychires.2010.11.008 21146190

[54] Waxman R, Fenton MC, Skodol AE, Grant BF, Hasin D. Childhood maltreatment and personality disorders in the USA: specificity of effects and the impact of gender. Personal Ment Health. 2014;8(1):30–41. 10.1002/pmh.1239 PMC3927226 24532553

[55] Griffin DW, Bartholomew K. Models of the self and other: Fundamental dimensions underlying measures of adult attachment. J Pers Soc Psychol. 1994;67(3):430.

[56] Baryshnikov I, Joffe G, Koivisto M, Melartin T, Aaltonen K, Suominen K, et al. Relationships between self-reported childhood traumatic experiences, attachment style, neuroticism and features of borderline personality disorders in patients with mood disorders. J Affect Disord. 2017:82–9. 10.1016/j.jad.2016.12.004 28024223

[57] Battle CL, Shea MT, Johnson DM, Zlotnick C, Zanarini MC, Sanislow CA, et al. Childhood maltreatment associated with adult personality disorders: findings from the collaborative longitudinal personality disorders study. J Personal Disord. 2004:193. 10.1521/pedi.18.2.193.32777 15176757

[58] Hareli S, Parkinson B (2008). What’s social about social emotions? J theory Soc Behav. Wiley Online Library.

[59] Gaher RM, Hofman NL, Simons JS, Hunsaker R (2013). Emotion regulation deficits as mediators between trauma exposure and borderline symptoms. Cogn Ther Res.

[60] Khosravi M (2020). Child maltreatment-related dissociation and its core mediation schemas in patients with borderline personality disorder. BMC Psychiatry.

[61] Zanarini WAA, Lewis RE, Reich RB, Vera SC, Marino MF, et al. Reported pathological childhood experiences associated with the development of borderline personality disorder. Am J Psychiatry. 1997;154(8):1101–6. 10.1176/ajp.154.8.1101 9247396

[62] Division (DCD) DC. What is child abuse or neglect? What is the definition of child abuse and neglect? [Internet]. HHS.gov. 13AD [cited 2022 Nov 3]. Available from: https://www.hhs.gov/answers/programs-for-families-and-children/what-is-child-abuse/index.html

[63] Carvalho Fernando S, Beblo T, Schlosser N, Terfehr K, Otte C, Lowe B, et al. Associations of childhood trauma with hypothalamic-pituitary-adrenal function in borderline personality disorder and major depression. Psychoneuroendocrinology. 2012;37(10):1659–68. 10.1016/j.psyneuen.2012.02.012 22444624

[64] Aase H, Sagvolden T. Moment-to-moment dynamics of ADHD behaviour. Behav Brain Funct. 2005;1(1):12. 10.1186/1744-9081-1-12 PMC1208851 16060963

[65] Harned MS, Korslund KE, Foa EB, Linehan MM. Treating PTSD in suicidal and self-injuring women with borderline personality disorder: development and preliminary evaluation of a dialectical behavior therapy prolonged exposure protocol. Behav Res Ther. 2012;50(6):381–6. 10.1016/j.brat.2012.02.011 PMC3348973 22503959

[66] Harned MS, Korslund KE, Linehan MM. A pilot randomized controlled trial of dialectical behavior therapy with and without the dialectical behavior therapy prolonged exposure protocol for suicidal and self-injuring women with borderline personality disorder and PTSD. Behav Res Ther. 2014;55:7–17. 10.1016/j.brat.2014.01.008 PMC3987949 24562087

[67] Pabst A, Schauer M, Bernhardt K, Ruf-Leuschner M, Goder R, Elbert T, et al. Evaluation of narrative exposure therapy (NET) for borderline personality disorder with comorbid posttraumatic stress disorder. Clin Neuropsychiatry. 2014;11(4):108–17.

[68] Steuwe C, Rullkötter N, Ertl V, Berg M, Neuner F, Beblo T, et al. Effectiveness and feasibility of narrative exposure therapy (NET) in patients with borderline personality disorder and posttraumatic stress disorder–a pilot study. BMC Psychiatry. 2016;16:254. 10.1186/s12888-016-0969-4 PMC4955150 27439618

[69] Linehan MM, Comtois KA, Ward-Ciesielski EF (2012). Assessing and managing risk with suicidal individuals. Cogn Behav Pract.

