# Supplementary material for: Differential associations between childhood maltreatment types and borderline personality disorder from the perspective of emotion dysregulation
Source: Borderline Personal Disord Emot Dysregul. 2023 Feb 6;10:4. doi: 10.1186/s40479-023-00210-7 (PMC9903452; doi:10.1186/s40479-023-00210-7)
Supplement: Supplementary file 1 — Additional file 1: Table 1. Demographic Differences in BPD Scores (BOR). Table 2. Demographic Differences in BPD Scores (PAIBOR). [file 40479_2023_210_MOESM1_ESM.docx]

Supplementary Tables

| Table 1 Demographic Differences in BPD Scores (BOR) | | | | | |
| --- | --- | --- | --- | --- | --- |
| **ANOVA Results** | | | | | |
|  | Df | Sum Sq | Mean Sq | F | P |
| Race | 2 | 142.70 | 71.35 | 3.47 | 0.03* |
| Hispanic | 1 | 0.40 | 0.42 | 0.02 | 0.89 |
| Marriage | 1 | 27.00 | 26.95 | 1.31 | 0.25 |
| Education | 6 | 122.60 | 20.44 | 0.99 | 0.43 |
| Employment | 4 | 186.30 | 46.57 | 2.27 | 0.07 |
| Sexual Orientation | 3 | 184.20 | 61.41 | 2.99 | 0.03* |
| Residuals | 126 | 2590.60 | 20.56 |  |  |
|  |  |  |  |  |  |
| **Tukey Post Hoc (Race and Sexual Orientation),95% CI** | | | | | |
|  | Difference | Lower | Upper | p |  |
| Multi v.s. Black | 5.33 | -0.97 | 11.63 | 0.11 |  |
| White v.s. Black | -1.29 | -3.30 | 0.72 | 0.29 |  |
| White v.s.Multi | -6.62 | -13.05 | -0.18 | 0.04 |  |
|  |  |  |  |  |  |
| Gay/lesbian/homosexual v.s. Bisexual | -2.37 | -6.57 | 1.83 | 0.46 |  |
| Heterosexual v.s. Bisexual | -2.49 | -5.20 | 0.23 | 0.09 |  |
| Not sure v.s. Bisexual | 2.77 | -5.93 | 11.47 | 0.84 |  |
| Heterosexual v.s. Gay/lesbian/homosexual | -0.12 | -3.71 | 3.48 | 1.00 |  |
| Not sure v.s. Gay/lesbian/homosexual | 5.14 | -3.88 | 14.16 | 0.45 |  |
| Not sure v.s. Heterosexual | 5.25 | -3.17 | 13.68 | 0.37 |  |

| Table 2 Demographic Differences in BPD Scores (PAIBOR) | | | | | |
| --- | --- | --- | --- | --- | --- |
| **ANOVA Results** | | | | | |
|  | Df | Sum Sq | Mean Sq | F | P |
| Race | 2 | 684 | 342.10 | 3.01 | 0.05 |
| Hispanic | 1 | 128 | 128.50 | 1.13 | 0.29 |
| Marriage | 1 | 11 | 11.30 | 0.10 | 0.75 |
| Education | 6 | 1005 | 167.60 | 1.47 | 0.19 |
| Employment | 4 | 1920 | 480.10 | 4.22 | 0.00* |
| Sexual Orientation | 3 | 166 | 55.40 | 0.49 | 0.69 |
| Residuals | 126 | 14331 | 113.70 |  |  |
|  |  |  |  |  |  |
| **Tukey Post Hoc (Employment),95% CI** | | | | | |
|  | Difference | Lower | Upper | p |  |
| No disabled-Homemaker | 19.60 | -9.92 | 49.12 | 0.36 |  |
| No not working-Homemaker | 1.41 | -19.88 | 22.71 | 1.00 |  |
| Yes full time-Homemaker | 2.75 | -18.69 | 24.20 | 1.00 |  |
| Yes part time-Homemaker | -3.35 | -24.59 | 17.90 | 0.99 |  |
| No not working-No disabled | -18.19 | -39.48 | 3.11 | 0.13 |  |
| Yes full time-No disabled | -16.85 | -38.29 | 4.60 | 0.20 |  |
| Yes part time-No disabled | -22.95 | -44.20 | -1.70 | 0.03 |  |
| Yes full time-No not working | 1.34 | -5.14 | 7.82 | 0.98 |  |
| Yes part time-No not working | -4.76 | -10.56 | 1.04 | 0.16 |  |
| Yes part time-Yes full time | -6.10 | -12.43 | 0.23 | 0.06 |  |
